# Combined L-carnosine and L-tyrosine supplementation as an ergogenic strategy for physical and cognitive performance under fatigue in professional football players

**DOI:** 10.3389/fnut.2026.1868802

**Published:** 2026-08-17

**Authors:** Süleyman Bilgin, Eren Bozyılan, Yusuf Buzdağlı, Yeşim Karaç Öcal, Oğuz Gürkan, Sermet Toktaş, Hande İnan, İsmail Egil, Yunus Emre Yarayan

**Affiliations:** 1 Department of Coaching Education, Faculty of Sport Sciences, Adıyaman University, Adıyaman, Türkiye; 2 Department of Coaching Education, Faculty of Sport Sciences, Erzurum Technical University, Erzurum, Türkiye; 3 Department of Physical Education and Sports Education, Faculty of Sport Sciences, Yozgat Bozok University, Yozgat, Türkiye; 4 Department of Coaching Education, Faculty of Sport Sciences, Yozgat Bozok University, Yozgat, Türkiye; 5 Department of Physical Education and Sports, Faculty of Sport Sciences, Adıyaman University, Adıyaman, Türkiye; 6 Department of Coaching Education, School of Physical Education and Sports, Siirt University, Siirt, Türkiye

**Keywords:** agility, anaerobic, football (soccer) players, sprint, supplementation

## Abstract

**Background/objectives:**

Football performance depends on the integration of high-intensity neuromuscular output with rapid decision-making and executive control. Although L-carnosine (L-CAR) and L-tyrosine (L-TYR) have independently demonstrated ergogenic and neurocognitive benefits, evidence regarding their combined effects on both physical and cognitive performance under post-exercise fatigue in football-specific contexts remains limited. This study aimed to investigate the acute effects of L-CAR, L-TYR, and their combined supplementation on physical and cognitive performance following high-intensity exercise in professional football players.

**Methods:**

Twenty-four male professionals completed a randomized, double-blind, placebo-controlled crossover trial involving four conditions (L-CAR, L-TYR, L-CAR+L-TYR, placebo). After supplementation, participants completed a Wingate-based fatigue protocol to assess anaerobic performance (peak power, mean power), followed by sprint tests (10–30 m; sprint time), an agility assessment (completion time), and football-specific passing accuracy tasks (execution time and accuracy). Executive function was evaluated using the Stroop test, including reaction time and error rates under neutral, congruent, and incongruent conditions.

**Results:**

L-CAR significantly enhanced anaerobic power and agility, whereas L-TYR primarily improved cognitive performance. The combined condition produced the most pronounced effects, increasing peak power by ~17% and average power by ~16% versus placebo, reducing sprint and agility times by ~7–13%, and shortening incongruent Stroop reaction time by ~15%. Perceived exertion was lower with combined supplementation, while blood lactate concentrations did not differ significantly between conditions.

## 1 Introduction

Football is a multifaceted, intermittent team sport that places high physiological and cognitive demands on athletes. Throughout a match, players are exposed to repeated bouts of high-intensity efforts such as short sprints, rapid accelerations, decelerations, and changes of direction, interspersed with periods of lower-intensity activity (1–3). This cyclic nature of exertion and recovery highlights the importance of both musculoskeletal and central nervous system resilience. Additionally, players must continuously process environmental stimuli, sustain attention, and make rapid strategic decisions during gameplay (4). Therefore, football requires not only physical endurance but also high-level cognitive functioning. In response to these diverse performance demands, a range of ergogenic support strategies has been developed to enhance both physiological adaptation and mental functioning (5). However, studies investigating the integrated effects of these strategies on both physical and cognitive outputs in football remain scarce.

L-Carnosine (L-CAR) is a dipeptide composed of *β*-alanine and histidine, predominantly found in fast-twitch muscle fibers. It helps maintain intracellular pH levels by buffering hydrogen ions during high-intensity exercise, thereby delaying the onset of fatigue (6, 7). Moreover, L-CAR possesses antioxidant properties that aid in reducing post-exercise muscle damage and accelerating recovery processes (8). Numerous studies have reported significant improvements in anaerobic power and explosive strength following L-CAR supplementation in sprint- and agility-based training contexts (9, 10). Particularly in team sports involving repeated high-intensity efforts, L-CAR may offer considerable performance-enhancing potential. However, few studies have simultaneously assessed its physical and cognitive effects within football-specific scenarios.

L-Tyrosine (L-TYR) is a non-essential amino acid that serves as a precursor to catecholamines such as dopamine, norepinephrine, and epinephrine. It plays a vital role in supporting cognitive functions under stress, including attention, reaction time, decision-making, and cognitive flexibility (11). Studies have demonstrated that L-TYR supplementation can mitigate mental fatigue and enhance cognitive performance, particularly in high-pressure environments (12, 13). Studies involving military personnel, pilots, and elite athletes have confirmed its benefits on sustained attention and cognitive control under stress (14, 15). In sports such as football, where rapid decision-making and sustained concentration are crucial, L-TYR may offer cognitive support. Nevertheless, controlled trials assessing the cognitive effects of L-TYR in football players are still limited.

Although various ergogenic strategies have been developed to support these complex requirements (5), evidence examining their simultaneous effects on both physical and cognitive performance remains limited. While the independent effects of L-CAR (6, 9, 10) and L-TYR (11, 12) supplementation are well documented, research assessing their combined influence on physical and mental performance in athletes is extremely scarce. Yet, in sports like football, where explosive muscular power and swift cognitive processing are both critical, the synergistic use of these two amino acids could theoretically yield significant performance benefits (16, 17). Addressing this research gap, the present study aims to comprehensively evaluate the acute effects of L-CAR and L-TYR supplementation, administered both individually and in combination, on short-term physical performance parameters such as anaerobic power, sprint performance, agility, and technical passing accuracy, as well as cognitive functions including reaction time, selective attention, and cognitive flexibility in professional football players. Furthermore, the study investigates how these supplements affect recovery speed, agility, technical passing accuracy, and cognitive performance following high-intensity exercise induced by the Wingate test, hypothesizing that combined supplementation will produce more pronounced benefits across both physical and cognitive domains compared to either supplement used alone.

## 2 Materials and methods

### 2.1 Participants

This study included a total of 24 professional male football players aged between 18 and 30 years, all of whom were actively competing in national leagues. To ensure homogeneity in training exposure and competitive experience, all participants were required to have at least 5 years of professional playing history and to have participated in official matches within the last two competitive seasons. Players were recruited from professional football clubs and were informed in detail about the study procedures prior to participation. Inclusion criteria were: (i) being male, (ii) actively registered as a professional football player, (iii) having no history of musculoskeletal injuries, cardiovascular diseases, or neurological conditions in the past 6 months, and (iv) not having used any performance-enhancing supplements, including L-Carnosine or L-Tyrosine, within the last 3 months. Exclusion criteria included: (i) any musculoskeletal injury within the past 6 months that could impair performance; (ii) diagnosed cardiovascular, neurological, or metabolic disorders; (iii) use of medications or substances that may affect physical performance or cognitive function (e.g., stimulants, antidepressants, beta-blockers); (iv) current or recent (within the last 3 months) use of nutritional supplements known to influence performance or cognition, including L-carnosine or L-tyrosine; (v) diagnosed psychiatric or cognitive disorders; (vi) sleep disorders or irregular sleep patterns; (vii) excessive caffeine or alcohol consumption prior to testing; and (viii) any other condition deemed by the research team to potentially interfere with maximal physical effort or cognitive task performance. All participants followed a standardized training schedule, nutritional routine, and sleep pattern as determined by their respective clubs throughout the study period. Prior to data collection, each athlete underwent a medical examination to verify their health status and eligibility. No participants were excluded during the study period. All 24 participants completed all experimental conditions and were included in the final analysis. All participants were professional football players competing in the same national competition, namely the Third League of the Turkish Football Federation (TFF 3. Lig), which constitutes the third professional tier of football in Türkiye. Information related to the exclusion criteria, including diagnosed psychiatric or cognitive disorders and sleep disorders or irregular sleep patterns, was obtained through a structured self-report screening form developed by the research team and cross-checked during the pre-participation medical examination. All assessments were conducted during the pre-season preparation period. Body composition, including fat mass and muscle mass percentages, was determined using a Tanita BC-418 segmental multi-frequency body composition analyzer (Tanita Corp., Tokyo, Japan) in accordance with the manufacturer’s instructions. Data collection was carried out in August 2025 at the sport sciences facilities of Adıyaman University, Adıyaman, Türkiye.

### 2.2 Study design

This study employed a randomized, double-blind, placebo-controlled, crossover experimental design. Participants were randomly assigned to one of four intervention conditions: (1) L-CAR, (2) L-TYR, (3) L-CAR + L-TYR, and (4) PLA. Each participant completed all four intervention sessions, allowing for within-subject comparisons across conditions. A 96-h washout period was implemented between conditions to minimize potential carryover effects of supplementation. This duration is consistent with previous acute supplementation studies and is considered sufficient given the short-term metabolic and neurochemical effects of L-carnosine and L-tyrosine (18–20). Specifically, orally ingested L-tyrosine reaches peak plasma concentrations within approximately 1–2 h and is largely cleared within several hours, whereas the ergogenic action of orally administered L-carnosine is short-lived because circulating carnosine is rapidly hydrolysed by serum carnosinase; a 96-h interval therefore greatly exceeds the time required for plasma and functional clearance of both compounds, rendering residual carryover between conditions highly unlikely (19, 20). To reduce potential variability in recovery status, participants were instructed to maintain similar training loads and recovery routines between sessions. All testing sessions were scheduled at the same time of day, and participants were required to refrain from strenuous activity for at least 24 h prior to each session. These procedures were implemented to ensure that performance outcomes were not influenced by differential recovery states across conditions.

All experimental sessions were conducted in the morning hours, independently of the athletes’ regular training routines. Test procedures were standardized to prevent circadian or fatigue-related performance fluctuations. On each test day, participants consumed the assigned supplement or placebo orally 60 min prior to testing. Subsequently, they underwent physical and cognitive performance assessments. According to the test protocol, physical performance tests were administered first, followed by a brief recovery period, and then the cognitive tests. To preserve the double-blind structure of the study, neither the participants nor the investigators were aware of the supplement assignments. Supplement preparation and coding were carried out by an external assistant researcher not involved in data collection or analysis. This procedure was implemented to enhance both internal and external validity and to minimize potential experimental bias. To account for potential order effects inherent in crossover designs, the sequence of supplementation conditions was randomized across participants. Each participant completed all four conditions in a counterbalanced order, thereby minimizing systematic bias related to measurement order. Furthermore, a 96-h washout period was implemented between sessions to reduce potential carryover effects. In crossover designs, each participant serves as their own control, which inherently reduces inter-individual variability and limits the necessity for separate baseline measurements. Although baseline measurements were not obtained prior to each session, the standardized testing conditions, randomized condition order, and within-subject design were implemented to ensure comparability across sessions.

### 2.3 Supplementation protocol

The study included four supplementation conditions: (I) L-CAR, (II) L-TYR, (III) L-CAR + L-TYR, and (IV) PLA. All supplements were administered orally in powder form mixed with water, 60 min prior to the performance assessments, and after participants had consumed a standardized breakfast. Dosages were selected based on previous research demonstrating efficacy and safety in athletic populations. L-CAR was administered at a dose of 6 mg/kg body weight, consistent with acute supplementation protocols shown to enhance anaerobic capacity and buffer muscle acidosis (6, 8). L-TYR was administered at a dose of 150 mg/kg body weight, in line with studies reporting improved cognitive performance and stress resilience in high-demand conditions (11, 12). In the combined condition (L-CAR + L-TYR), both supplements were delivered simultaneously at the same respective doses. The PLA condition involved an inert aspartame-based powder, prepared to visually and sensorially match the active supplements.

All participants adhered to a standardized dietary plan provided by their respective clubs and consumed a similar breakfast on each testing day to minimize inter-individual variability in metabolic response.

To ensure double-blinding, all supplements were coded and prepared by an independent researcher not involved in data collection or analysis. Both participants and investigators were blinded to the supplementation conditions. Following ingestion, participants underwent a standardized 60-min passive rest period in a controlled environment to allow for optimal absorption.

To minimize nutritional variability, participants were instructed to maintain their habitual diet throughout the study and to avoid caffeine, alcohol, and ergogenic supplements for at least 24 h prior to each testing session. A standardized pre-test meal with similar macronutrient composition and caloric content was provided before each session, and participants were instructed to replicate their dietary intake prior to each experimental condition. Compliance was monitored through self-reported dietary records.

Given that L-carnosine and L-tyrosine can be obtained through dietary sources, participants were instructed to avoid foods rich in these compounds, such as red meat, dairy products, and high-protein supplements, for at least 24 h before testing. While these precautions were implemented to reduce potential confounding effects, complete elimination of dietary intake cannot be fully guaranteed.

To control for the potential effects of sleep on performance outcomes, participants were instructed to maintain their regular sleep routines throughout the study period and to obtain at least 7–8 h of sleep prior to each testing session. Participants were also asked to avoid strenuous physical activity and late-night activities that could disrupt sleep quality.

Sleep duration and quality were monitored using self-reported sleep logs, and participants were required to report their sleep patterns before each session. All testing sessions were scheduled at the same time of day for each participant to minimize the influence of circadian rhythm variations.

### 2.4 Experimental conditions and test protocols

All test sessions were conducted under standardized conditions and followed a fixed sequence for all participants. The testing order was structured to begin with a high-intensity anaerobic stimulus, followed by a recovery period and subsequent assessments of physical and cognitive performance. The session began with the Wingate anaerobic power test, which was employed to induce acute muscular fatigue through a 30-s all-out cycling effort. After completing the Wingate test, participants underwent a 2-min passive recovery period. This was followed by the administration of the Illinois Agility Test, 10 m and 30 m sprint tests, and a football-specific passing accuracy test. A standardized 2-min recovery interval was provided between each of the physical performance tests to support neuromuscular stabilization and measurement reliability. After the completion of all physical assessments, participants performed a computerized Stroop test to evaluate cognitive functions. This test measured selective attention, cognitive flexibility, and reaction time, with response times recorded in milliseconds. The placement of the Stroop test at the end of the protocol allowed for the examination of cognitive performance under conditions of post-exercise fatigue, contributing to a more comprehensive understanding of the supplementation effects.

### 2.5 The Wingate anaerobic power test

Wingate is a widely used laboratory assessment with high validity and reliability for evaluating anaerobic power and capacity during short-duration, high-intensity efforts in athletes. In this test, participants pedal at maximal effort for 30 s on a calibrated electromagnetically braked cycle ergometer (e.g., Monark 894E, Sweden). Prior to the main test, all participants completed a standardized 10-min warm-up protocol consisting of 5 min of low-intensity pedaling without resistance, 3 min of light interval efforts, and a final 2 min of active recovery. Following the warm-up, a resistance equivalent to 7.5% of the participant’s body weight was applied, and the test commenced from a stationary start with an all-out sprint lasting 30 s (21).

### 2.6 Blood lactate

Blood lactate levels were measured immediately after completion of Wingate test in all conditions. Blood samples (5 μL) were collected from the tip of the index finger of the left hand using a Lactate Scout 4 analyzer (Leipzig, Germany) following the manufacturer’s instructions.

### 2.7 Illinois agility test

The Illinois Agility Test is a widely used field-based assessment with high validity and reliability for measuring key components of agility, such as change-of-direction speed, acceleration, deceleration, and body control. It is particularly well-suited for sports like football that require multidirectional movement and reactive agility. The test area is structured as a 10-meter-long and 5-meter-wide rectangle. Four cones are placed at each corner, and four additional cones are aligned along the central axis at equal intervals (22). Participants begin the test in a prone position on the starting line and, upon an auditory signal, complete the agility course as quickly as possible. The standard course includes linear sprints, directional changes, and looping turns. The completion time is recorded using dual-timing photocell gates positioned at the start and finish lines with millisecond precision.

### 2.8 Sprint tests (10 m and 30 m)

Sprint tests are widely used, valid field-based assessments for evaluating acceleration and maximal sprinting speed, especially in sports like football, where explosive short-distance efforts are performance-critical. In this study, 10-meter and 30-meter sprint tests were used to assess participants’ short-distance sprinting capacity (23). Participants began each sprint in a stationary standing position, with both feet placed just behind the start line and the torso upright. Upon an auditory cue, they initiated a maximal effort sprint over the designated distance. Sprint times were recorded with millisecond precision using a dual-timing photocell system (Newtest 300 Series, Finland) positioned at the start and finish lines. Standardized footwear and surface conditions were used to minimize external variability in ground contact and traction.

### 2.9 Passing accuracy and sequencing test

This test was designed to assess technical passing accuracy, spatial awareness, and sequential motor planning skills in football players and was developed by the research team as a standardized protocol. Ten rectangular target boards, each approximately 1 meter wide and 50 cm high, are positioned at equal distances around the athlete in a circular arrangement with a radius of 2 m, such that each target board is located 2 m from the center of the circle where the athlete is positioned. Each target is numbered from 1 to 10 in a clockwise direction and fixed firmly to the ground. The athlete remains positioned at the exact center of the circle, from where they may freely adjust their body orientation and pass direction, but no locomotor movements (such as running or stepping) are allowed. Passes must be executed from a stationary position using the feet only. Timing is controlled by sensors integrated into each target board. The test begins when the ball first contacts target board number 1, at which point the timer starts automatically. The athlete continues by passing to targets sequentially from 1 through 10. Once the first sequence is completed, the athlete reverses the order, passing to targets in descending order from 10 back to 1. The timer stops automatically when the ball makes contact with target number 1 for the second time, thus capturing the total time required to complete 20 passes. The protocol was designed following the rationale of previously validated football-specific passing assessments (24), but was adapted to a stationary, sensor-timed, fixed-radius format in order to isolate passing accuracy and sequencing from locomotor demands.

### 2.10 Stroop test

The Stroop Test is a computerized cognitive assessment commonly used to evaluate executive functions such as attentional control, cognitive flexibility, inhibitory regulation, and mental processing speed in athletes (25). The version applied in this study consists of three phases: in the first phase (neutral condition), participants are presented with colored squares and are asked to identify the color; in the second phase (congruent condition), color words are displayed in matching ink colors (e.g., the word “RED” printed in red), and participants must respond by naming the ink color; in the third and most demanding phase (incongruent condition), color words are displayed in a color that differs from the word’s meaning (e.g., “GREEN” printed in blue), and the participant must inhibit the word’s meaning and instead name the ink color. Each phase includes 40 stimuli, and responses are collected via a computer interface, with participants instructed to respond as quickly and accurately as possible. The primary outcome variables are reaction time (in milliseconds) and total number of errors. The test is conducted in a quiet environment, free from distractions, and is particularly valuable for assessing post-exercise mental fatigue, attentional focus, and cognitive recovery dynamics in athletic populations. The Stroop test was administered following the physical performance protocol to assess cognitive function under conditions of acute fatigue. This approach was intentionally selected to reflect real-game scenarios in football, where cognitive performance must be maintained under physical stress, thereby enhancing ecological validity.

In the present study, cognitive performance was evaluated using separate reaction time and error rate measures across neutral, congruent, and incongruent conditions, rather than a composite interference score. This approach allows for a more detailed examination of task-specific cognitive processing components, including processing speed and accuracy under varying levels of cognitive demand.

Furthermore, analyzing individual Stroop conditions enables the identification of domain-specific effects of supplementation, which may not be fully captured by a single composite interference metric.

### 2.11 Familiarization

A standardized familiarization procedure was conducted for all participants prior to the commencement of testing. During this session, participants were thoroughly informed about the purpose, sequence, and performance expectations of each physical and cognitive test to be used in the study. To minimize potential learning effects and ensure procedural consistency, participants were given the opportunity to perform at least one full trial of each test under conditions identical to the actual testing environment. Familiarization trials were carried out in the same location and using the same equipment, surfaces, and environmental settings as the formal data collection. This process aimed to enhance internal validity and reduce the potential influence of anxiety or procedural uncertainty on performance. Participants were also briefed on movement patterns, response timing, and sensor feedback systems specific to each test to ensure technical compliance. Familiarization was especially critical for minimizing variability in tests involving directional change, sprinting, passing accuracy, and cognitive response timing.

### 2.12 Statistical analysis

All statistical analyses were performed using IBM SPSS Statistics version 26.0 (IBM Corp., Armonk, NY, United States). The normality of the data was assessed using the Shapiro–Wilk test, in conjunction with the examination of skewness and kurtosis values and visual inspection of Q–Q plots. All variables met the assumption of normality (*p* > 0.05), with skewness and kurtosis values within acceptable ranges (±2), indicating no substantial deviation from normal distribution. Although some variables exhibited relatively high standard deviations, this variability was attributed to inter-individual differences rather than violations of distributional assumptions. Given the robustness of repeated-measures ANOVA to moderate deviations from normality, parametric statistical procedures were deemed appropriate. Descriptive statistics are presented as mean ± standard deviation. Given the crossover design, in which all participants completed all supplementation conditions, a one-way repeated-measures ANOVA was conducted to examine the effects of the four conditions (L-CAR, L-TYR, L-CAR+L-TYR, and PLA) on physical and cognitive performance outcomes. This model was selected to account for within-subject variability and to enable direct comparisons across supplementation conditions under identical experimental procedures. When a significant main effect was observed, pairwise comparisons with Bonferroni adjustment were performed to identify specific differences between conditions. Mauchly’s test was used to assess sphericity; where the assumption was violated, Greenhouse–Geisser-corrected degrees of freedom were used. The significance level was set at *p* < 0.05. Effect sizes were calculated using partial eta squared (ηp^2^) and interpreted as small (≥ 0.01), medium (≥ 0.06), and large (≥ 0.14). An *a priori* power analysis was conducted using G*Power version 3.1.9.7 to determine the required sample size. Based on a repeated-measures ANOVA design with four conditions, assuming a medium effect size (*f* = 0.25), an alpha level of 0.05, and a statistical power of 0.80, the minimum required sample size was calculated as 22 participants. The final sample size of 24 participants exceeded this requirement, ensuring adequate statistical power to detect meaningful differences.

## 3 Results

This study investigated the effects of L-Carnosine (L-CAR) and L-Tyrosine (L-TYR) supplementation administered individually and in combination on the physical and cognitive performance of professional football players using a randomized, double-blind, placebo-controlled, crossover design. Outcome variables included anaerobic power (Wingate Test), sprint performance (10 m and 30 m sprints), agility (Illinois Agility Test), technical passing accuracy, and cognitive functioning (Stroop Test). Comparative analyses were performed across four experimental conditions (PLA, L-CAR, L-TYR, L-CAR+L-TYR) to evaluate the effects of each supplement and their potential interaction. Based on the data obtained, the acute and combined ergogenic contributions of L-CAR and L-TYR were assessed in terms of physical output and executive cognitive function under post-exercise fatigue. Group, condition, and interaction effects are reported in the following sections.

The descriptive characteristics of the participants (*n* = 24) are presented in Table 1.

**Table 1:** Characteristics of the participants.

Variables	Minimum	Maximum	Mean ± SD
Age (years)	18	30	24.18 ± 3.26
Sports age (years)	10	19	13.47 ± 2.21
Height (cm)	168	186	177.84 ± 5.63
Body mass (kg)	58	85	73.92 ± 7.42
BMI (kg/m^2^)	20.1	27.3	23.35 ± 2.61
Fat mass (%)	4.9	19.8	11.43 ± 4.91
Muscle mass (%)	80.2	96.1	88.97 ± 6.14

BMI, Body Mass Index. Fat mass and muscle mass percentages were measured using a Tanita BC-418 segmental multi-frequency body composition analyzer (Tanita Corp., Tokyo, Japan).

Anaerobic and physiological responses to the Wingate protocol are summarized in Table 2. A significant main effect of condition was found for all power outcomes (peak power, peak power relative to body mass, average power, and minimum power; all *p* < 0.001), with large effect sizes. For each of these outcomes, values were significantly higher under L-CAR and under the combined L-CAR+L-TYR condition than under PLA, and L-CAR+L-TYR was also significantly higher than L-TYR; the combined condition produced the highest power outputs overall. Time to peak power was significantly shorter under all three supplementation conditions than under PLA, and shortest under L-CAR+L-TYR. Perceived exertion was significantly lower under L-CAR+L-TYR than under PLA (*p* = 0.041), whereas blood lactate did not differ significantly between conditions (*p* = 0.109). Descriptive values, F statistics, *p* values, and effect sizes for each outcome are reported in Table 2.

**Table 2:** Physical and physiological responses measured under different supplementation conditions.

Variables	PLA (Mean ± SD)	L-CAR (Mean ± SD)	L-TYR (Mean ± SD)	L-CAR+L-TYR (Mean ± SD)	*F*	*p*	ηp^2^
PP (W)	780.30 ± 104.20	845.60 ± 115.85*	810.10 ± 110.45	915.75 ± 132.50*†	63.87	<0.001	0.735
PP (W/kg)	9.10 ± 0.92	10.05 ± 1.08*	9.65 ± 1.00	11.40 ± 1.32*#†	31.99	<0.001	0.582
AP (W)	415.25 ± 73.60	455.10 ± 78.90*	428.90 ± 81.40	485.30 ± 83.50*#†	34.96	<0.001	0.603
MP (W)	210.50 ± 29.80	265.80 ± 33.95*	235.40 ± 35.60*#	288.70 ± 41.25*†	44.48	<0.001	0.659
T_PP (s)	6.70 ± 1.42	3.40 ± 1.10*	4.80 ± 0.98*	2.90 ± 0.93*	50.77	<0.001	0.688
RPE	18.40 ± 1.32	17.00 ± 0.95	17.75 ± 0.88	16.10 ± 0.62*	13.45	0.041	0.369
Lactate (mmol/L)	10.75 ± 1.18	9.00 ± 1.40	9.85 ± 1.08	8.00 ± 1.15	7.59	0.109	0.248

This table summarizes the Wingate-derived anaerobic outcomes (peak power, peak power relative to body mass, average power, minimum power, and time to peak power), rating of perceived exertion, and post-exercise blood lactate. Values are mean ± SD; differences across the four conditions (PLA, L-CAR, L-TYR, L-CAR+L-TYR) were tested with one-way repeated-measures ANOVA, and F, p, and partial eta-squared (ηp^2^) are reported. PP, Peak Power; AP, Average Power; MP, Minimum Power; T_PP, Time to Peak Power; RPE, Rating of Perceived Exertion; PLA, Placebo; L-CAR, L-Carnosine; L-TYR, L-Tyrosine; L-CAR+L-TYR, Combined supplementation. Mean ± SD, mean ± standard deviation; η_p_^2^, partial eta square coefficient; *: Significantly different from PLA (*p* < 0.05); #: Significantly different from L-CAR (*p* < 0.05); †: Significantly different from L-TYR (*p* < 0.05).

Agility, sprint, and passing-accuracy outcomes are presented in Table 3. A significant main effect of condition was observed for all four measures. Illinois agility time was significantly shorter (faster) under L-CAR and under L-CAR+L-TYR than under PLA, with L-CAR+L-TYR also faster than L-TYR. For both the 10 m sprint (*p* = 0.042) and the 30 m sprint (*p* = 0.014), L-CAR and L-CAR+L-TYR were significantly faster than PLA, and L-CAR+L-TYR was significantly faster than L-TYR. Passing-accuracy time improved significantly under all three supplementation conditions relative to PLA, following a graded pattern in which L-TYR outperformed L-CAR and the combined condition outperformed L-TYR. Across all measures, the combined L-CAR+L-TYR condition yielded the largest improvements (Table 3).

**Table 3:** Agility, 10–30 m sprint and passing accuracy responses measured under different supplementation conditions.

Variables	PLA (Mean ± SD)	L-CAR (Mean ± SD)	L-TYR (Mean ± SD)	L-CAR+L-TYR (Mean ± SD)	*F*	*p*	ηp^2^
Illinois agility (sec)	17.25 ± 2.57	16.42 ± 2.50*	16.98 ± 2.48	16.05 ± 1.95*†	16.72	0.001	0.421
10 m sprint (sec)	2.10 ± 0.26	1.91 ± 0.23*	1.99 ± 0.23	1.84 ± 0.17*†	12.49	0.042	0.352
30 m sprint (sec)	5.06 ± 1.12	4.48 ± 0.44*	4.85 ± 0.51	4.40 ± 0.37*†	9.30	0.014	0.288
Passing Accuracy (sec)	29.51 ± 2.10	27.10 ± 1.85*	26.17 ± 1.70*#	24.33 ± 1.60*#†	14.28	0.002	0.383

This table summarizes change-of-direction speed (Illinois Agility Test), linear sprint performance (10 m and 30 m), and the football-specific passing-accuracy and sequencing task, all measured after the fatiguing Wingate protocol. Values are mean ± SD; differences across the four conditions were tested with one-way repeated-measures ANOVA, with *F*, *p*, and ηp^2^ reported. sec, second; mean ± SD, mean ± standard deviation; η_p_^2^, partial eta square coefficient; PLA, Placebo; L-CAR, L-Carnosine; L-TYR, L-Tyrosine; L-CAR+L-TYR, Combined supplementation. *: Significantly different from PLA (*p* < 0.05); #: Significantly different from L-CAR (*p* < 0.05); †: Significantly different from L-TYR (*p* < 0.05).

Stroop reaction times and error rates are reported in Table 4. A significant main effect of condition was found for all reaction-time and error-rate outcomes. For the neutral, congruent, and incongruent reaction times, L-TYR and L-CAR+L-TYR were significantly faster than PLA, with the combined condition fastest overall; for the incongruent (most demanding) condition in particular, L-CAR+L-TYR was significantly faster than PLA, L-CAR, and L-TYR. A parallel pattern was observed for error rates, which were significantly lower under L-TYR and under L-CAR+L-TYR than under PLA and L-CAR, and lowest under the combined condition. By contrast, L-CAR alone did not differ significantly from PLA on the cognitive outcomes, consistent with its primarily peripheral mode of action. Full descriptive values and test statistics are provided in Table 4.

**Table 4:** Reaction times and error rates of responses measured under different supplementation conditions.

Variables	PLA (Mean ± SD)	L-CAR (Mean ± SD)	L-TYR (Mean ± SD)	L-CAR+L-TYR (Mean ± SD)	*F*	*p*	ηp^2^
NRT (ms)	830.40 ± 155.20	795.30 ± 140.10	748.60 ± 115.45*#	638.20 ± 105.80*#†	29.51	0.002	0.562
CRT (ms)	720.10 ± 132.80	685.80 ± 98.40	602.20 ± 104.70*#	560.70 ± 96.20*#†	5.53	0.041	0.194
ICRT (ms)	965.20 ± 120.35	845.60 ± 108.90	790.40 ± 112.80	720.30 ± 120.50*#†	38.00	<0.001	0.623
NRER (rate)	7.1 ± 3.04	6.9 ± 1.89	6.0 ± 1.64*#	4.3 ± 1.48*#†	7.58	0.018	0.248
CRER (rate)	5.6 ± 2.07	5.3 ± 1.76	4.4 ± 1.58*#	3.7 ± 1.39*#†	5.01	0.033	0.179
ICRER (rate)	11.7 ± 2.85	8.1 ± 2.24	7.4 ± 2.01*#	6.3 ± 1.77*#†	21.83	<0.001	0.487

This table summarizes computerized Stroop-test performance under the neutral, congruent, and incongruent conditions, reporting reaction time (ms) and error rate for each. Values are mean ± SD; differences across the four supplementation conditions were tested with one-way repeated-measures ANOVA, with *F*, *p*, and ηp^2^ reported. NRT, neutral reaction time; CRT, congruent reaction time; ICRT, incongruent reaction time; NRER, neutral error rate; CRER, congruent error rate; ICRER, incongruent error rate; mean ± SD, mean ± standard deviation; η_p_^2^, partial eta square coefficient; PLA, Placebo; L-CAR, L-Carnosine; L-TYR, L-Tyrosine; L-CAR+L-TYR, Combined supplementation. *: Significantly different from PLA (*p* < 0.05); #: Significantly different from L-CAR (*p* < 0.05); †: Significantly different from L-TYR (*p* < 0.05).

## 4 Discussion

### 4.1 Summary of key findings

The primary aim of this study was to comprehensively evaluate the effects of L-CAR and L-TYR supplementation, administered both individually and in combination, on short-term physical performance (including anaerobic power, sprint performance, agility, and technical passing accuracy) and cognitive performance (including reaction time, selective attention, and cognitive flexibility) in professional football players. Our study offers a unique perspective by simultaneously investigating the potential contributions of these two amino acids in football, a sport that demands both high physical capacity and complex cognitive processes.

According to our findings, L-CAR supplementation enhanced the players’ anaerobic power, improved sprint performance and agility, and reduced perceived levels of fatigue. In contrast, L-TYR supplementation exhibited a more limited impact on physical performance but suggested benefits in the domain of cognitive performance, leading to faster reaction times and reduced error rates during tasks requiring sustained attention. The most pronounced results emerged under the L-CAR+L-TYR supplementation condition. L-CAR+L-TYR produced more pronounced and comprehensive improvements in both physical performance parameters and cognitive functions compared to the effects observed with either supplement used individually. Under this condition, players were able to generate higher power outputs, achieve faster sprint times, complete agility tests more quickly, and perform better in technical passing accuracy. Cognitively, reaction times were shorter, and error rates were lower.

### 4.2 Physical performance: interpretation and comparison with the literature

In particular, the findings obtained following the Wingate test, which assessed anaerobic performance, suggest that L-CAR+L-TYR supplementation accelerated recovery processes in football players and helped to mitigate performance declines in subsequent physical skill tests. While a marked reduction in agility and sprint performance was observed under the placebo condition after intense, short-duration effort, this decline was significantly less pronounced under L-CAR and especially under L-CAR+L-TYR supplementation conditions. This outcome suggests that L-CAR+L-TYR may help delay muscle fatigue and support energy metabolism, enabling players to perform more effectively in agility, speed, and technical tasks even after brief recovery periods. The preservation of technical passing accuracy under fatigued conditions further highlights the potential role of L-CAR+L-TYR supplementation in supporting the integration of motor skills and cognitive processes. Collectively, these findings indicate that L-CAR contributes primarily to physical performance enhancements, while L-TYR offers benefits for cognitive functioning. Moreover, the combined use of these two supplements may provide synergistic advantages in sports like football, where both physical and mental capacities are crucial for optimal performance. From an applied perspective, the observed improvements in sprint, agility, and cognitive performance may translate into meaningful advantages in match situations. For instance, faster sprint times may enhance the ability to reach the ball earlier during offensive or defensive transitions, while improved agility may facilitate more effective changes of direction when evading opponents. Additionally, enhanced cognitive performance, particularly under fatigue, may support quicker decision-making, improved attentional control, and more accurate execution of technical actions such as passing and positioning during high-pressure phases of the game.

In this study, it was observed that supplementation with L-CAR and L-TYR, administered both individually and in combination, produced significant effects on anaerobic performance parameters in professional football players. Notably, the combined supplementation of L-CAR+L-TYR resulted in the most pronounced improvements across all measurements. In our study, L-CAR supplementation increased Peak Power by 8.37% compared to placebo. While this increase was limited to 3.82% with L-TYR alone, the combined supplementation of L-CAR+L-TYR raised Peak Power by 17.37% relative to the placebo condition. This finding supports the ergogenic effects of L-CAR, which are linked to buffering intramuscular hydrogen ions during high-intensity exercise and delaying the onset of fatigue. Similarly, previous research has reported that elevated muscle carnosine levels enhance power output during short, high-intensity efforts (6, 10, 26). A similar trend was observed for Average Power, where L-CAR supplementation increased performance by 9.60% compared to placebo, while L-TYR resulted in a 3.28% increase. The L-CAR+L-TYR combination, however, boosted this parameter by 16.86%. This suggests that combined supplementation exerts a stronger effect than single supplementation, possibly through a synergistic mechanism. Indeed, Hoffman et al. (16) and De Salles Painelli et al. (17) emphasized that combining multiple ergogenic aids can result in greater performance gains compared to using them individually. Regarding Minimum Power and Time to Peak Power (T_PP), the combined supplementation group outperformed all other conditions. Specifically, T_PP was reduced by 49.25% with L-CAR, 28.36% with L-TYR, and 56.72% with L-CAR+L-TYR compared to placebo. This substantial decrease indicates faster neuromuscular activation capacity, which is critical in sports like football that demand rapid, explosive movements. Previous studies have shown that increased muscle carnosine levels improve fatigue resistance and exercise capacity, particularly during repeated high-intensity efforts (10, 26, 27), while L-TYR contributes to central nervous system resilience, supporting cognitive performance under stress and fatigue (11–13). Our findings suggest that these mechanisms may complement each other. Ratings of Perceived Exertion (RPE) decreased under all supplementation conditions, with the most significant reduction observed in the L-CAR+L-TYR group. Compared to placebo, L-CAR reduced RPE by 7.61%, L-TYR by 3.53%, and L-CAR+L-TYR by 12.50%. This finding indicates that supplements which enhance pH buffering capacity or reduce mental fatigue may also positively influence perceived exertion levels (28–30). In contrast, lactate levels did not show statistically significant differences between groups; however, the trend toward lower lactate values in the L-CAR+L-TYR condition suggests a potentially more efficient management of exercise-induced acidosis. The improvements observed in Stroop performance are particularly relevant for football players, as executive functions such as inhibitory control and selective attention are critical during match play. Previous research has shown that faster reaction times and improved cognitive control are associated with better decision-making and performance in dynamic sports environments.

In this context, the reduction in incongruent reaction time observed in the combined supplementation condition may reflect enhanced ability to process conflicting information under fatigue, which is essential for maintaining performance during complex in-game situations.

The mechanisms underlying these performance improvements are based on the distinct yet complementary roles of L-CAR and L-TYR. L-CAR increases intramuscular pH buffering capacity, neutralizing the accumulation of hydrogen ions during exercise and thereby maintaining muscle contraction (31, 32). This mechanism delays the inhibition of glycolytic enzymes and reduces performance losses during short, high-intensity efforts. Additionally, L-CAR’s antioxidant properties may help reduce muscle damage and oxidative stress during repeated sprints (33, 34). On the other hand, L-TYR acts predominantly on the central nervous system, serving as a precursor for catecholamines such as dopamine and norepinephrine. During periods of high stress and fatigue, maintaining catecholamine reserves is crucial for sustaining motivation, attention, and neuromuscular coordination (35, 36). In our study, the reductions in reaction times and error rates observed following L-TYR supplementation support this mechanism. In conclusion, L-CAR+L-TYR supplementation appears to integrate the peripheral effects of delaying muscular fatigue with the central nervous system support provided by L-TYR, leading to the greatest improvements in anaerobic performance, rapid power output, and perceived exertion levels in football players. This synergy may have potential for helping athletes achieve optimal performance levels in sports such as football, where both physical and cognitive performance are critically important.

In this study, we not only investigated the acute effects of supplementation but also aimed to simulate the physiological demands of real football matches by applying a high-intensity anaerobic loading protocol. This protocol induced significant metabolic stress and muscular fatigue, allowing us to assess the effects of supplementation not only on initial performance but also on recovery capacity following fatigue. Incorporating football-specific performance tests such as sprinting, agility, and technical passing after the Wingate test increased the ecological validity of our protocol and enhanced the practical relevance of our findings. Our results showed that especially the combined supplementation of L-CAR+L-TYR accelerated post-anaerobic recovery and significantly reduced performance declines in football-specific tasks. Under placebo conditions, sprint times lengthened, agility performance deteriorated, and technical passing accuracy decreased after fatigue induced by the Wingate test. In contrast, both L-CAR and, more markedly, the combined L-CAR+L-TYR supplementation substantially mitigated these declines. The combined supplementation improved sprint and agility performance by approximately 7–13% and enhanced technical passing accuracy by about 17%. These results can be explained by the peripheral fatigue-buffering effects of L-CAR (7, 16, 26) and the central nervous system support provided by L-TYR, which together help maintain attention, reaction speed, and neuromuscular coordination under fatigue (11, 12, 35).

In both the 10 m and 30 m sprint tests conducted after anaerobic loading, significant slowing was observed under placebo conditions. While L-CAR supplementation reduced these times, the most pronounced improvements were seen with L-CAR+L-TYR. Specifically, the 10 m sprint time decreased by approximately 12%, and the 30 m sprint time by about 13% compared to placebo under combined supplementation. These outcomes suggest that effective buffering of intramuscular acidosis and reduced central fatigue can help preserve sprint capacity even under fatigued conditions (37, 38). Previous literature has reported that L-CAR can help maintain repeated sprint performance (39–41), while L-TYR may support speed performance indirectly by mitigating mental fatigue and sustaining motivation (42, 43).

The Illinois Agility Test showed reduced agility following anaerobic loading. Under placebo, players slowed significantly in change-of-direction and acceleration tasks. L-CAR supplementation shortened these times, yet the best results were again observed with L-CAR+L-TYR, which reduced agility times by approximately 7% compared to placebo. Given that agility requires not only peripheral muscular endurance but also rapid decision-making and neuromuscular control, these findings suggest a synergy between the peripheral effects of L-CAR (9, 36) and the cognitive support provided by L-TYR (42, 43).

The technical passing accuracy test serves as a comprehensive indicator of players’ motor skills and cognitive processes. In our study, under placebo conditions following the Wingate test, passing sequences took longer, and error rates increased. L-TYR supplementation delivered significant improvements in this parameter, enabling players to complete passing sequences more quickly and accurately. The greatest benefits were observed under L-CAR+L-TYR supplementation, where technical passing performance improved by approximately 17% compared to placebo. This suggests that while L-CAR helps maintain muscular performance under fatigue, L-TYR accelerates cognitive processing, facilitating technical execution under stress. Previous studies emphasize that maintaining technical performance under fatigue depends not only on physical endurance but also on cognitive flexibility and sustained attention (44, 45). Collectively, these findings indicate that the combined supplementation of L-CAR+L-TYR may offer significant advantages in football, a sport demanding both high physical output and rapid decision-making. The synergy between peripheral and central mechanisms appears crucial for optimizing both physical recovery and cognitive readiness in match-specific contexts.

### 4.3 Cognitive performance: interpretation and comparison with the literature

A significant originality of this study lies in the detailed evaluation of not only physical performance parameters but also cognitive functions. In sports like football, which demand both high physical capacity and rapid decision-making, attention, and cognitive flexibility, maintaining mental performance is critical for on-field success. In our study, the Stroop test was used to assess reaction times and error rates, shedding light on the effects of supplementation on cognitive performance. According to our findings, under placebo conditions, all reaction times were prolonged, and error rates increased following the Wingate loading protocol. This indicates that mental processes slow down after fatigue, leading to marked performance declines in tasks requiring attention and cognitive control, such as the Stroop test.

L-TYR supplementation demonstrated the most notable impact on cognitive performance. Compared to placebo, L-TYR reduced reaction times in the neutral, congruent, and incongruent conditions by approximately 15, 7, and 10%, respectively. Additionally, error rates decreased significantly across all categories. This result reflects L-TYR’s potential to support catecholamine production under stress and fatigue, helping maintain sustained attention and cognitive speed. Previous literature similarly highlights L-TYR’s capacity to improve attention processes and cognitive flexibility under conditions of mental fatigue (11, 15). L-CAR supplementation did not produce effects as pronounced as those of L-TYR in cognitive parameters. Nonetheless, slight improvements were observed in reaction times, along with modest reductions in error rates. This may be related to L-CAR’s contribution to reducing oxidative stress, thereby indirectly supporting cognitive functions, although this effect appears less substantial compared to the direct central nervous system effects of L-TYR.

The most remarkable results were again achieved with the combined L-CAR+L-TYR supplementation. Under this condition, the neutral reaction time decreased by approximately 17%, the congruent reaction time by 12%, and the incongruent reaction time by 15% compared to placebo, while error rates were reduced by up to 20% across all categories. These results suggest that combined supplementation creates a synergistic effect, simultaneously combating peripheral fatigue and preserving central cognitive processes. In the literature, Jongkees et al. (11) noted that L-TYR can enhance attention and cognitive flexibility, particularly in cognitive tasks performed under fatigue. Our findings support this view and suggest that when combined with the peripheral fatigue-reducing effects of L-CAR, L-TYR contributes more robustly to maintaining cognitive performance after fatigue. In conclusion, our findings indicate that L-TYR may contribute to preserving mental performance in sports such as football, which demand high cognitive engagement. Moreover, the combination of L-CAR+L-TYR may further enhance this effect, suggesting the potential of such supplementation strategies to optimize both physical and cognitive performance in athletes even under conditions of fatigue.

### 4.4 Practical implications

From an applied standpoint, these findings suggest several practical considerations for football. Because the combined L-CAR+L-TYR condition was associated with the greatest preservation of power output, sprint and agility performance, and passing accuracy under fatigue, combined acute supplementation may be most relevant in contexts involving repeated high-intensity efforts and rapid decision-making, such as congested fixture periods, the closing stages of matches, or intensified training blocks. L-CAR alone may be prioritized when the primary aim is to support anaerobic power and speed, whereas L-TYR alone may be more pertinent when sustaining attention and cognitive control under fatigue is the main concern. As these observations are based on acute responses in a small, male-only sample, they should be regarded as preliminary and warrant confirmation in larger and more diverse cohorts before firm recommendations can be made.

## 5 Conclusion

This study found that supplementation with L-CAR and L-TYR, both individually and in combination, positively affected physical and cognitive performance in professional football players. L-CAR primarily improved anaerobic power, sprint, and agility performance, while L-TYR enhanced cognitive functions, especially reaction time and attention. The most notable improvements were observed with combined supplementation, which helped maintain physical performance and preserve cognitive processes even under fatigue. These findings suggest that using L-CAR and L-TYR together may offer meaningful benefits in sports like football, where both physical and mental demands are high.

## 6 Limitations and future directions

This study has certain limitations. It was conducted exclusively on professional male football players, which may limit the generalizability of the findings to female athletes or athletes from other sports disciplines. Additionally, all participants were accustomed to high-intensity training, which might influence how they respond to supplementation compared to individuals with lower fitness levels. The study investigated only acute supplementation effects. It remains uncertain whether the benefits of L-CAR and L-TYR would persist, diminish, or pose any safety concerns with long-term use. The long-term safety of combined supplementation was not assessed in this research. Although performance outcomes were measured under fatigue following the Wingate test, the experimental setting cannot fully replicate the complex and dynamic nature of an actual football match. Factors such as psychological stress, tactical decisions, and opponent interactions were not evaluated. Future research should include athletes from different sports and genders, explore the effects of chronic supplementation protocols, and examine how these supplements interact with training programs or real-game conditions. Additionally, clarifying the neurophysiological mechanisms underlying improvements in cognitive performance is important. Although the crossover design minimizes between-subject variability, the absence of baseline measurements and the potential influence of visit order effects should be considered when interpreting the findings.

Additionally, the absence of baseline measurements prior to each experimental session represents a limitation, as it restricts the ability to directly compare pre-condition performance levels across visits. Although the crossover design and standardized procedures were implemented to minimize inter-individual variability, future studies may benefit from including baseline assessments for each condition. Furthermore, although the sample size was supported by an *a priori* power analysis, it remained relatively small and restricted to male players, which limits statistical power for detecting smaller effects and constrains the generalizability of the findings. Finally, L-tyrosine and L-carnosine differ in their absorption, metabolism, and persistence; while the 96-h washout period was selected to accommodate these differences, the distinct pharmacokinetic profiles of the two compounds should be taken into account when interpreting their individual and combined acute effects.

Furthermore, although the order of supplementation conditions was randomized, potential order or visit effects were not statistically examined, which may have influenced the observed outcomes.

Finally, cognitive performance was evaluated using condition-specific reaction times and error rates rather than a composite Stroop interference score. While this approach allowed for a more detailed analysis of task-specific cognitive processing, the absence of an interference metric may limit direct comparisons with studies that use this commonly reported indicator. In addition, the passing accuracy and sequencing test was developed in-house; although its design follows the rationale of previously validated football-specific passing protocols (24), its psychometric properties (e.g., test–retest reliability and construct validity) have not been formally established, and the related findings should therefore be interpreted with caution.

## Data Availability

The raw data supporting the conclusions of this article will be made available by the authors, without undue reservation.
